# In Memoriam: Karen Foster (1955–2022)

**DOI:** 10.3201/eid2808.220746

**Published:** 2022-08

**Authors:** Byron Breedlove

**Affiliations:** Centers for Disease Control and Prevention, Atlanta, Georgia, USA

**Keywords:** In memoriam, memoriam, Karen Foster, Emerging Infectious Diseases, Morbidity and Mortality Weekly Report

Karen Lynn Foster, who worked as a technical writer-editor and subject matter expert for *Emerging Infectious Diseases* from 2009 until early 2022, died on March 10, 2022, at age 66. Throughout her time with the journal, Karen was known for her exemplary knowledge of language, editing, grammar, and science and for her ability to handle tough assignments and deadlines.

Born in Quonset Point, Rhode Island, USA, the oldest of 6 children, Karen graduated cum laude from Longwood College, Farmville, Virginia, USA, where she received a Bachelor of Arts in English. Karen earned a Master of Arts in Creative Writing from Hollins College, Roanoke, Virginia, and also completed postgraduate work in English at Vanderbilt University, Nashville, Tennessee, USA.

In 1982, she joined the then–Centers for Disease Control as an assistant editor for the *Morbidity and Mortality Weekly Report*, followed by a stint as a writer-editor for what was then the Center for Infectious Diseases. During 1988−2000, Karen served as managing editor of *MMWR*. She worked another 5 years as a technical writer-editor in the National Center for Environmental Health/Agency for Toxic Substances & Disease Registry before retiring from CDC in 2005. During the next phase of her career, which also encompassed her time with *Emerging Infectious Diseases*, Karen worked as a freelance writer/editor and editorial consultant in public health and epidemiology. Karen is among the editors of the second edition of *Law in Public Health Practice* (2006) and served as one of the managing editors of the *CDC Field Epidemiology Manual* (2019).

Karen’s career and accomplishments are all the more remarkable in that she overcame a serious visual handicap to excel in a profession that required close and careful reading. Her colleagues recall Karen not only for her exacting editorial skills and high standards but also for having a terrific sense of humor and formidable wit. 

Apart from work, Karen’s true passion was raising, showing, and breeding English Springer Spaniels. She was a charter member of the Chattahoochee English Springer Spaniel Club of Greater Atlanta and was also a member of English Springer Rescue America and the English Springer Spaniel Field Trial Association.

Karen leaves behind many colleagues who, during the course of 4 decades, worked with her, worked for her, and learned from her. Some may be interested to know that shortly before her passing, Karen finished writing her first novel, which her family hopes to have published in the near future. We offer our condolences to her surviving family members and friends.

**Figure:**
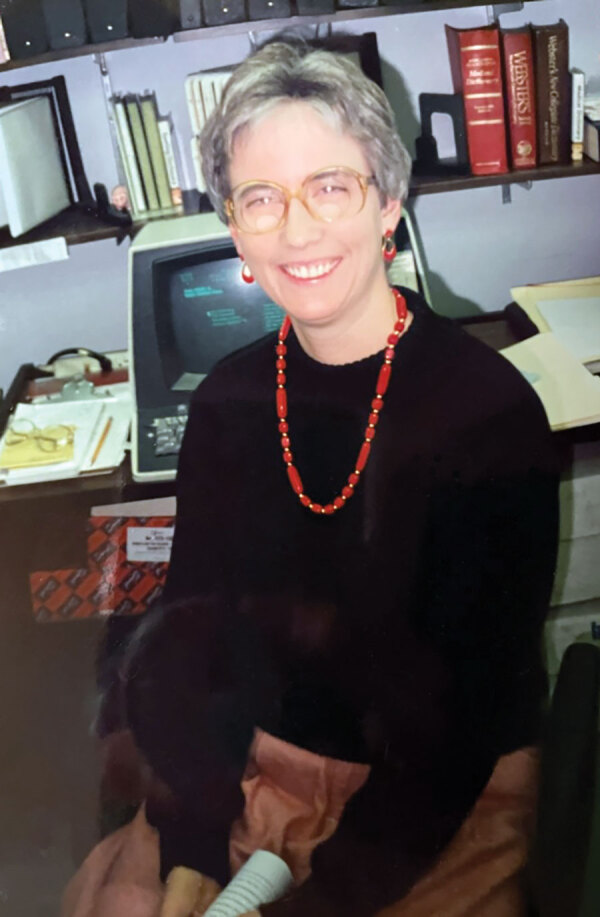
Karen Foster (1955–2022). Image courtesy of the Foster family.

